# Barriers and limitations for undergoing mammography screenings among Kuwaiti women (aged 40–69) attending primary health care centers

**DOI:** 10.1186/s12875-025-02971-2

**Published:** 2025-09-29

**Authors:** Omar Alfailakawi, Hussain Alostath, Asmaa Aldubian, Al-Ghala Alsaleh, Ghadeer Alsughayer, Alaa Alshamran, Ali Ashkanani, Hussein Kamal, Haneen Mohammad, Mohammad Murad, Duaa Hasan, Mohamed Hany Shehata

**Affiliations:** 1 Medical Student at the College of Medicine and Health Sciences, Arabian Gulf University, Manama, Kingdom of Bahrain; 2 Family Physician, Governmental Hospitals, Manama, Kingdom of Bahrain; 3 Department of Family and Community Medicine, College of Medicine and health Sciences, Arabian Gulf University, Manama, Kingdom of Bahrain; 4 Family medicine Department, Faculty of Medicine, Helwan University, Cairo, Egypt

**Keywords:** Breast cancer screening, Mammography, Kuwait, Barriers, Health equity

## Abstract

**Background:**

Breast cancer is the leading cancer among women in Kuwait, yet mammography screening (MS) uptake remains suboptimal despite the Kuwait National Mammography Screening Program (KNMSP). This study identifies barriers to MS adherence among Kuwaiti women aged 40–69, focusing on sociocultural, knowledge-related, and systemic factors.

**Methods:**

A cross-sectional study was conducted across five primary healthcare centers in Kuwait (June–August 2023) using semi-structured interviews with 201 women. Data were analyzed via SPSS to assess awareness, attitudes, and screening uptake.

**Results:**

Only 25.4% of participants underwent MS within the past two years, with 52.2% never screened. Despite 91.5% being aware of screening programs, 84.6% exhibited poor overall awareness. Key barriers included fear of pain (77.6%) and physical discomfort (76.1%). Younger women (40–49 years) and employed individuals had significantly lower uptake (*p* = 0.001 and *p* = 0.004, respectively). Women invited to screen were 61.4% more likely to participate (*p* < 0.001). Trust in screening programs strongly correlated with adherence (*p* < 0.001), while conflicting medical advice reduced uptake (*p* = 0.03).

**Conclusion:**

Low MS uptake persists due to fear, discomfort, and gaps in targeted education, despite program awareness. Younger and employed women require tailored interventions. Recommendations include culturally resonant campaigns addressing modesty concerns, pre-screening counseling to alleviate pain-related anxiety, and proactive invitation systems via employer partnerships or automated reminders. Future studies should employ population-based sampling and qualitative.

**Supplementary Information:**

The online version contains supplementary material available at 10.1186/s12875-025-02971-2.

## Introduction

Globally, breast cancer is a significant public health challenge, accounting for 12.5% of all new annual cancer cases [1, 2]. The burden of breast cancer is higher in Kuwait as it is the most prevalent cancer, representing 39.5% of all cancers diagnosed in women and 20.6% of cancers among both sexes [1]. This incidence rate among Kuwaiti women is more than double the global average, denoting an urgent need for targeted interventions. Furthermore, evidence suggests that malignant breast cancers diagnosed in Kuwait tend to be more aggressive when compared to other regions [3].

There is collective evidence on the critical importance of early detection. According to the American Cancer Society, when breast cancer is detected at a localized stage, the 5-year relative survival rate is 99%. However, this rate drops to 86% for regional disease and decreases even more to 31% for distantly metastasized cancer [4]. Early detection and treatment do not only improve survival rates but also significantly decrease the financial burden on patients and healthcare systems. The cost of treatment escalates in proportion with the cancer stages, rising from an average of $60,000 for stage 0 to over $136,000 for stage IV [5]. Therefore, timely diagnosis is necessary for improving patient outcomes and mitigating economic costs [6–13].

Mammography is internationally recognized as the gold standard for breast cancer screening due to its proven ability to detect lesions before they become palpable, its favourable risk-benefit profile, and its reasonable cost and sensitivity [14, 15]. In response to the growing burden of breast cancer, Kuwait launched the Kuwait National Mammography Screening Program (KNMSP) in 2014 to promote early screening of breast cancer [16].

Despite this national initiative, participation remains critically low. The KNMSP reported a participation rate of only 7.8% among 14,773 eligible asymptomatic women [16]. This trend of suboptimal compliance is reflected across the Gulf Cooperation Council (GCC) countries, where nations including Saudi Arabia, Oman, and the UAE also report screening participation rates below 15% of the target population [17–21].

A substantial body of literature identifies key factors affecting mammography uptake. Higher educational levels, increased awareness, and higher socioeconomic status are positively correlated with screening uptake [22, 23]. Conversely, numerous barriers prevent women from undergoing screening. Both international and regional studies consistently cite fear of a cancer diagnosis, lack of physician recommendation, insufficient knowledge about breast cancer, lack of social support, and mistrust in the healthcare system as significant obstacles [24–26]. A qualitative study in Kuwait further identified barriers related to personal factors, medical services delivery, and the social environment [27].

However, there remains a clear research gap concerning a comprehensive, community-level investigation into these factors in Kuwait. The interplay between knowledge, awareness, attitudes, and their collective impact on mammography uptake has not been sufficiently studied within the local context.

This study, therefore, aims to explore the relationships between awareness levels, attitudes, and mammography screening uptake among women in Kuwait. To achieve this, the study is designed to utilize a semi-structured questionnaire to pursue several key objectives: (1) to assess the awareness levels of Kuwaiti women regarding mammography screening.; (2) to evaluate attitudes, perceptions, and beliefs influencing Kuwaiti women’s willingness to participate in and the actual uptake of mammography screening; (3) to identify demographic and socioeconomic factors (e.g., age, education, income, marital status) associated with disparities in mammography screening uptake; (4) to quantify the association between specific knowledge gaps regarding mammography guidelines (such as recommended starting age and frequency) and screening uptake; (5) to investigate systemic and individual barriers (e.g., fear of diagnosis, logistical challenges, lack of physician recommendation) and facilitators (e.g., social support, prior positive screening experiences) affecting screening adherence.

The findings will provide an evidence-based foundation for developing targeted awareness campaigns and strategic interventions to enhance the effectiveness of the KNMSP, with the ultimate goal of reducing breast cancer mortality through timely detection.

## Materials and methods

### Study design and population

A cross-sectional study was conducted among Kuwaiti women aged 40–69 years attending primary healthcare centers. This age range was selected as it aligns with the target demographic for the Kuwait National Mammography Screening Program (KNMSP) and international screening guidelines.

### Sample size calculation

The sample size was determined using the single-population proportion formula [28]:

$$n=Z^2p\left(1=p\right)/d^2$$

Where:

- Z = 1.96 (for a 95% confidence level).
- *p* = 0.147 (proportion of mammography uptake).
- d = 0.05 (5% margin of error).

The proportion (p) was estimated at 0.147 (14.7%) based on a report on screening rates available during the study’s design phase [18]. While a more recent KNMSP-specific report indicates a lower uptake of 7.8% for asymptomatic screening [16], our calculation based on the 14.7% figure ensured a robust sample size sufficient for the study’s objectives.

Substituting the values:

$$n=\left(0.05\right)2\left(1.96\right)20.147\left(1-0.147\right)\approx193$$

To account for potential non-response or incomplete data, this number was inflated by approximately 4%, resulting in a final target sample size of 201 participants.

### Sampling and recruitment

A multi-stage purposive and convenience sampling method was employed for this study. In the first stage, three of Kuwait’s governorates (Al-Asimah, Hawally, and Al-Ahmadi) were purposefully selected. This selection was based on logistical accessibility and to ensure geographic diversity representing Kuwait’s varied urban and suburban landscape; the Capital Governorate (Al-Asimah) is the nation’s most densely populated urban center, Hawally is a high-density residential and commercial district, and Al-Ahmadi encompasses a mix of suburban communities.

In the second stage, a total of five primary healthcare centers with high patient volume were selected from these governorates: three centers from the Capital Governorate, one from the Hawalli Governorate, and one from the Al-Ahmadi Governorate. At these sites, participants were recruited using a convenience sampling approach, chosen for its practicality and for enabling efficient access to the target population within the study’s timeframe.

#### Eligibility criteria

Participants were required to meet the following criteria:

- Kuwaiti women aged 40–69 years.
- No personal history of breast cancer.
- Not currently experiencing active breast-related symptoms.

#### Recruitment process and sample distribution

The recruitment process began with clinic nurses and staff identifying and referring potentially eligible women to the research team during their routine visits. Researchers then confirmed eligibility using a brief checklist and obtained written informed consent from all willing participants before administering the questionnaire.

Recruitment continued across the five centers until the target sample size of 201 participants was reached. The target of recruiting approximately 40 participants per center was a pragmatic strategy to ensure a balanced and manageable data collection workload across the sites. Proportional representation of the population was addressed not by the quota per center, but by the strategic selection of more centers (three) from the most populous region (the Capital Governorate). This design ensured that a larger proportion of the total sample (120 participants, or ~ 60%) was drawn from the capital, reflecting its higher population density relative to the Hawalli and Al-Ahmadi governorates (which contributed ~ 20% of the sample each).

Finally, we acknowledge that the primary limitation of convenience sampling is the potential for selection bias, which may limit the generalizability of the findings to all women in Kuwait. To mitigate this bias, this multi-site approach across three diverse governorates was implemented to capture a more heterogeneous sample and a broader cross-section of attendees than a single-site study would allow.

### Study instrument and data collection

Data were collected via face-to-face interviews using a semi-structured questionnaire developed by the research team.

#### Questionnaire validation

The instrument underwent a three-stage validation process to ensure its reliability and validity for the study population:

1. Development: Items were generated based on an extensive literature review of existing validated questionnaires on breast cancer screening and were adapted for the local context.
2. Content Validity: The draft questionnaire was reviewed by an expert panel, including an epidemiologist, a public health specialist, and two primary care physicians, to assess the relevance, clarity, and comprehensiveness of the items.
3. Pilot Testing: The revised questionnaire was pilot-tested on 10 women from the target population who were not included in the final sample. Feedback on cultural appropriateness and question clarity was used to produce the final version of the instrument.

The final questionnaire included the following key sections:

1. Sociodemographic Characteristics. This section collected data on participants’ age, marital status, educational background, employment status, and income level.
2. Mammography Screening Uptake. Participants’ self-reported screening history was categorized based on KNMSP guidelines.
  Good Uptake: Last mammogram within the past 2 years.
  Moderate Uptake: Last mammogram done over 2 years or those who were not sure about the timing of their last mammogram.
  Poor Uptake: Included women had never undergone a mammogram.
3. Awareness Assessment Awareness was evaluated using four questions covering key knowledge areas: the availability of screening programs in Kuwait, the indications for screening, the recommended age to start, and the appropriate frequency. Based on the number of correct responses, participants were classified into “Good Awareness” or “Poor Awareness” groups using the median score of the sample as an objective, data-driven cutoff.
4. Attitudes and Barriers to Screening. This section assessed barriers using ten items on a 4-point Likert scale (Strongly Disagree, Disagree, Agree, Strongly Agree). A 4-point scale was deliberately used as a “forced choice” design to encourage respondents to express a definitive stance.

For scoring, responses were dichotomized to simplify the data into a summative score representing the overall burden of barriers. “Disagreement” responses were assigned 2 points, while “Agreement” responses were assigned 4 points (with reverse-coding for positive statements). This resulted in a total barrier score for each participant.

Due to the lack of a previously validated instrument with established cutoffs for this population, a post-hoc, data-driven approach was used to categorize attitudes. The cutoff of ≤ 26 was statistically derived, representing approximately one standard deviation (SD = 4) below the sample mean score of 31.6. This method was chosen to systematically identify participants with substantially more positive attitudes (i.e., fewer perceived barriers) compared to the average respondent in our study.

### Statistical analysis

was performed using IBM SPSS Statistics for Windows (Version 28). Descriptive statistics were used to summarize all participant characteristics. Continuous data, specifically participant age, were expressed as mean ± standard deviation (SD). All categorical data including sociodemographic variables, awareness levels, and screening uptake categories were summarized as frequencies and percentages.

The Chi-square (χ²) test was employed to determine the statistical significance of associations between the categorical independent variables and the screening uptake outcome. For all statistical tests, a *p*-value of < 0.05 was considered significant.

## Results

Table 1 demonstrates the demographic data of the participants. The participants’ ages were between 40 and 69, with a mean age of 51.83 ± 8.75. Almost half of the study population were in the age group 40–49 (48.3%). Regarding marital status, most of the women (73.1%) were married, and only 9 (4.5%) were single. More than half of the study population (58.7%) had an educational level of a bachelor’s degree or higher. More than half (54.70%) of the participants were employed.

**Table 1:** Sociodemographic data of participants

Factor	Subgroup	*n* (%)
Governorate	Al-Asimah	80 (39.8%)
	Hawally	80 (39.8%)
	Al-Ahmadi	41 (20.4%)
Age Group	40–49	97 (48.3%)
	50–59	61 (30.3%)
	60–69	43 (21.4%)
Marital Status	Single	9 (4.5%)
	Married	147 (73.1%)
	Divorced	26 (12.9%)
	Widowed	19 (9.5%)
Education Level	Primary or Secondary	16 (8.0%)
	High School or Diploma	67 (33.3%)
	Bachelor’s degree	101 (50.2%)
	Postgraduate	17 (8.5%)
Occupation	Employed	110 (54.7%)
	Unemployed	91 (45.3%)

**Fig. 1:**
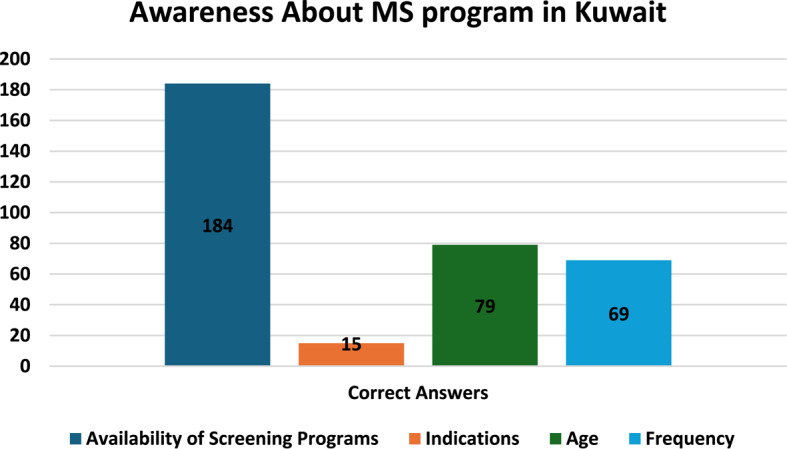
Participants’ awareness about MS program in Kuwait

As shown in Fig. 1. More than one third of the study population 79 women (39.3%) knew the correct age to start MS, while 69 (34.3%) knew its recommended frequency, and Only 15 (7.5%) knew the correct indications of when to perform it. On the contrary, the vast majority (91.5%) of women were aware of the availability of screening programs in Kuwait, regardless of their uptake.

**Fig. 2:**
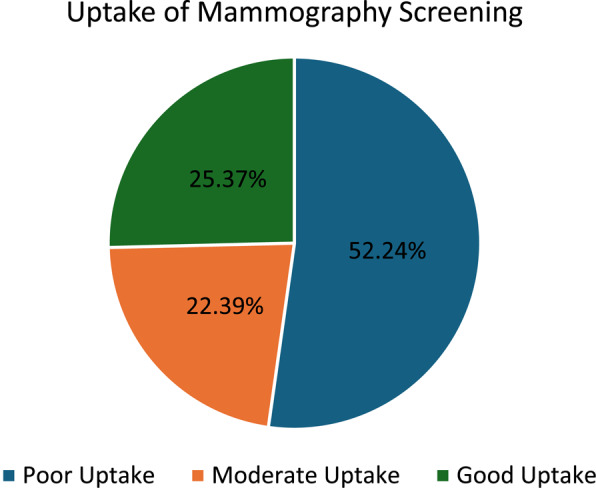
Overall uptake of MS

Out of the total population, we categorized 105 patients (52.2%) as having Poor Uptake, indicating that most participants in this study have never had a mammography screening in their lifetime. In contrast, 45 patients (22.3%) showed Moderate Uptake, which indicates they had a mammography screening but not within the last 2 years. Meanwhile, 51 patients (25.3%) demonstrated Good Uptake, representing those whose last mammogram was conducted within the past two years (Fig 2).

As for the effect of several sociodemographic variables on the mammography screening uptake, Table 2 shows that Al-Asimah governorate had the highest proportion of women with good mammography screening uptake, while Hawalli governorate had the lowest. Age was significantly associated with uptake (*p* = 0.001). Women aged 40–49 had the lowest uptake (67% poor uptake), while those aged 50–59 had the highest proportion of good uptake (37.7%). Uptake was also low among women aged 60–69 (with only 12 having good uptake). While widowed women had the highest percentage of good uptake (36.8%), marital status was not significantly associated with screening uptake. Employment status, however, was significantly associated with uptake (*p* = 0.004), with only 19.1% of employed women demonstrating good uptake.

**Table 2:** Uptake of mammography screening in comparison with sociodemographic data

Factor	Subgroup	Poor Uptake *n* (%)	Moderate Uptake *n* (%)	Good Uptake *n* (%)	*p*-Value (Chi-square test)
Governorate	Al-Asimah	41 (51.3%)	14 (17.5%)	25 (31.3%)	0.288
	Hawally	42 (52.5%)	23 (28.8%)	15 (18.8%)	
	Al-Ahmadi	22 (53.7%)	8 (19.5%)	11 (26.8%)	
Age Group	40–49	65 (67.0%)	16 (16.5%)	16 (16.5%)	0.001*
	50–59	16 (26.2%)	16 (26.2%)	23 (37.7%)	
	60–69	13 (30.2%)	13 (30.2%)	12 (27.9%)	
Marital Status	Single	6 (66.7%)	1 (11.1%)	2 (22.2%)	0.56
	Married	78 (53.1%)	31 (21.1%)	38 (25.9%)	
	Divorced	14 (53.8%)	8 (30.8%)	4 (15.4%)	
	Widowed	7 (36.8%)	5 (26.3%)	7 (36.8%)	
Education Level	Illiterate AND Primary or Secondary	7 (43.8%)	6 (37.5%)	3 (18.8%)	0.693
	High School or Diploma	37 (55.2%)	15 (22.4%)	15 (22.4%)	
	Bachelor’s degree	51 (50.5%)	22 (21.8%)	28 (27.7%)	
	Postgraduate	10 (58.8%)	2 (11.8%)	5 (29.4%)	
Occupation	Employed	69 (86.3%)	20 (25.0%)	21 (19.1%)	0.004*
	Unemployed	36 (45.0%)	25 (31.3%)	30 (33.0%)	

*Statistical significance

Table 3 describes the relation between uptake of MS and awareness about MS. While a high proportion of the 201 women surveyed (91.5%) were aware of the availability of mammography screening programs in Kuwait, this awareness did not translate into significantly higher uptake. Of the 31 women with good awareness, only 8 (25.8%) demonstrated good uptake, compared to 43 of 170 (25.3%) with poor awareness. However, knowledge of screening frequency and the recommended starting age for mammography were significantly associated with uptake. Among the 69 women who knew the correct screening frequency, 31.4% had good uptake and 51.1% had moderate uptake (*p* = 0.025). Furthermore, women with good uptake were more likely to know the recommended starting age (54.9%) compared to those with poor uptake, of whom 66.7% were unaware of the recommended age (*p* = 0.03).

**Table 3:** Uptake of mammography screening in relation to awareness about mammography screening

Factor	Answer	Poor Uptake *n* (%)	Moderate Uptake *n* (%)	Good Uptake *n* (%)	*p*-Value
Availability of screening programs in Kuwait	No	8 (7.6%)	7 (15.6%)	2 (3.9%)	0.112
	Yes	97 (92.4%)	38 (84.4%)	49 (96.1%)	
Indications of Breast Cancer screenings	Wrong Answer	93 (88.6%)	43 (95.6%)	50 (98.0%)	0.074
	Correct Answer	12 (11.4%)	2 (4.4%)	1 (2.0%)	
Age to Start Mammography	Wrong Answer	70 (66.7%)	29 (64.4%)	23 (45.1%)	0.03*
	Correct Answer	35 (33.3%)	16 (35.6%)	28 (54.9%)	
Frequency of Mammograms	Wrong Answer	75 (71.4%)	22 (48.9%)	35 (68.6%)	0.025*
	Correct Answer	30 (28.6%)	23 (51.1%)	16 (31.4%)	
Overall Awareness	Poor Awareness	90 (85.7%)	37 (82.2%)	43 (84.3%)	0.862
	Good Awareness	15 (14.3%)	8 (17.8%)	8 (15.7%)	

*Statistical significance

Table 4 describes the perceived barriers preventing women from undergoing mammography screening. Participants most frequently reported Fear of pain during the test (77.6%) and Physical discomfort of being touched (76.1%) as barriers. We found that the least prominent barrier was a lack of trust in screening programs, with only 24.4% of the population considering it a barrier.

**Table 4:** Perceived barriers of undergoing mammography screening among uptake groups

Factor		Poor Uptake *n* (%)	Moderate Uptake *n* (%)	Good Uptake *n* (%)	*p*-Value
Physical Discomfort	Disagree	23 (47.9%)	11 (22.9%)	14 (29.2%)	0.744
	Agree	82 (53.5%)	34 (22.2%)	37 (24.1%)	
Fear of Pain	Disagree	19 (42.2%)	12 (26.7%)	14 (31.1%)	0.31
	Agree	86 (55.1%)	33 (21.1%)	37 (23.7%)	
Received Conflicting Advice	Disagree	51 (44.7%)	27 (23.6%)	36 (31.5%)	0.03*
	Agree	54 (62.0%)	18 (20.6%)	15 (17.2%)	
Predetermined Course of BC	Disagree	38 (50.0%)	20 (26.3%)	18 (23.6%)	0.578
	Agree	67 (53.6%)	25 (20.0%)	33 (26.4%)	
Fear of Bad Results	Disagree	58 (46.7%)	29 (23.3%)	37 (29.8%)	0.103
	Agree	47 (61.0%)	16 (20.7%)	14 (18.1%)	
Unsure What to Expect of Mammograms	Disagree	34 (43.5%)	19 (24.3%)	25 (32.0%)	0.117
	Agree	71 (57.7%)	26 (21.1%)	26 (21.1%)	
Harmful Radiation	Disagree	37 (52.1%)	14 (19.7%)	20 (28.1%)	0.709
	Agree	68 (52.3%)	31 (23.8%)	31 (23.8%)	
Feeling of Obligation	Disagree	72 (53.7%)	33 (24.6%)	29 (21.6%)	0.194
	Agree	33 (49.2%)	12 (17.9%)	22 (32.8%)	
Trusts Screening Programs	Disagree	37 (75.5%)	9 (18.3%)	3 (6.1%)	< 0.001*
	Agree	68 (44.7%)	36 (23.6%)	48 (31.5%)	
More Important Problems	Disagree	50 (48.5%)	22 (21.3%)	31 (30.0%)	0.285
	Agree	55 (56.1%)	23 (23.4%)	20 (20.4%)	

*Statistical significance

Analysis of perceived barriers to mammography screening uptake revealed two significant associations. Women with low uptake were significantly more likely to report a lack of trust in screening programs (75.5%, *p* < 0.001) and to have received conflicting advice regarding mammography (62%, *p* = 0.03).

**Fig. 3:**
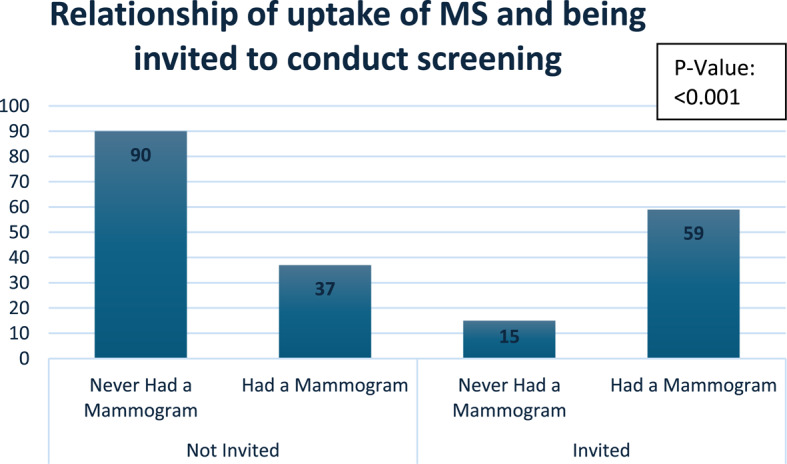
Relationship of uptake of Mammography screening and being invited to conduct screening

As shown in Fig 3 of the 96 women who underwent mammography, 59 (61.4%) reported receiving an invitation to do so. Women who did not receive an invitation were significantly less likely to have had a mammogram; 85.7% of women in the poor uptake group reported not receiving an invitation (*p* < 0.001).

## Discussion

Of course. After a comprehensive review of the provided text and all the previous reviewer comments, I have synthesized the different versions into a single, cohesive, and polished Discussion section.

This final version ensures consistency, improves the logical flow, and directly integrates the responses to all reviewer feedback.

### Final polished discussion section

This study aimed at exploring the barriers preventing Kuwaiti women aged 40–69 from adhering to mammography screening. Participants who reported proper uptake of mammography screening were 25.4% of the studied population. The findings also revealed that despite high awareness of available programs (91.5%), over half (52.2%) of participants exhibited poor uptake, underscoring a stark disconnect between knowledge and action. This aligns with global patterns where a combination of structural, psychosocial, and systemic barriers often impedes screening adherence [26, 29–31]. The predominance of fear-related barriers—such as pain (77.6%) and physical discomfort (76.1%)—mirrors findings from similar populations [32], suggesting that emotional and cultural apprehensions may outweigh awareness campaigns.

The 25.4% uptake rate observed in this study marks a potential improvement over earlier Kuwaiti reports [16, 18]. It is important to interpret this comparison with caution, as metrics may differ (e.g., adherence to guidelines in our study versus ‘ever screened’ in others). However, potential contributing factors for this apparent increase could include a heightened health consciousness among women attending primary healthcare centers, where data were collected, as well as evolving public health messaging. The alignment with recent Malaysian data [33] further underscores the role of temporal trends, such as global breast cancer advocacy efforts.

The persistence of 52.2% poor uptake (never screened) nonetheless signals that established barriers require targeted intervention. Poor uptake was significantly increased among employed women (19.1% uptake vs. 33.0% in unemployed, *p* = 0.004), which suggests a potential parallel with findings in Palestine where being busy hindered working women’s participation [34]. This may be due to logistical barriers such as inflexible work schedules and lack of time, a factor identified in other regional studies. It underscores the need for workplace wellness programs and employer partnerships to facilitate screening access.

Poor uptake was also significantly associated with younger participants in the current study. This is consistent with regional and international studies that reported higher uptake levels of mammography screening in older participants [25, 35]. Tailored campaigns for younger cohorts are critical, as delayed screening diminishes early detection benefits [36].

Interestingly, we did not find a significant association between screening uptake and education level or governorate. The lack of an educational gradient could suggest that our sample, being recruited from primary healthcare centers, may have a uniformly higher level of health literacy than the general population. Similarly, the absence of regional disparities could be interpreted as a positive indicator of the KNMSP’s success in achieving equitable program reach across the selected urban and suburban areas. However, we acknowledge that the reliability of some comparisons, particularly for variables with small subgroups like marital status, is limited, and these findings should be interpreted with caution.

A striking result was the poor uptake among women who were never invited by their doctors to perform mammography screening (85.7%) while 61.4% of screened women received formal invitations, with a statistically significant difference (*p* < 0.001). This aligns with the findings of [25, 37] as well as global evidence [38, 39] and highlights KNMSP’s untapped potential to leverage structured outreach, such as SMS reminders or community health worker networks, to bridge the invitation gap.

A central finding of this study is the significant paradox between awareness and action. While the vast majority of women (91.5%) knew that screening programs existed in Kuwait, this did not translate into higher uptake; in fact, 84.5% of the sample demonstrated poor functional awareness regarding screening specifics, mirroring findings in Saudi Arabia [40]. This “awareness-action gap” is further evidenced by the lack of a statistically significant association between simply being aware of the program’s existence and actually participating. The explanation for this discrepancy lies in the critical distinction between general and specific knowledge. While general awareness had no impact (only 25.8% of well-informed women demonstrated good uptake*)*, specific, actionable knowledge was a significant predictor of behavior. For instance, knowing the correct screening frequency (*p* = 0.025) and the recommended starting age (*p* = 0.03) were both significantly associated with higher screening uptake. These results align with literature from multiple studies emphasizing that specific, rather than general, knowledge drives behavior change [41, 42]. This also aligns with foundational behavioral science principles, such as the Health Belief Model, which posit that specific knowledge that informs a person’s perceived risk and benefits is a more powerful driver of behavior than general awareness [43, 44].

Ultimately, our data suggest that this awareness-action gap is sustained because even when women possess specific knowledge, their decision-making is more powerfully influenced by immediate emotional and systemic barriers. The most prominent barriers identified were fear of pain (77.6%) and physical discomfort (76.1%). Furthermore, a lack of trust in screening programs (*p* < 0.001) and receiving conflicting medical advice (*p* = 0.03) were significantly associated with poor uptake. This is similar to the findings of multiple studies [26, 37] Therefore, our findings indicate that future interventions must evolve beyond general awareness campaigns. To be effective, strategies must focus on mitigating these specific, powerful barriers—building patient-provider trust, standardizing medical advice, and addressing the deep-seated fears related to the screening procedure itself—to successfully bridge the gap between what women know and what they do.

Regarding barriers, our study examined 10 main items. Fear of pain during the test (77.6%) and physical discomfort of being touched (76.1%) were the most prominent barriers among the women surveyed reflecting cultural sensitivities around bodily exposure and medicalized touch, consistent with Saudi Arabian studies where 68% of women cited modesty concerns as a deterrent [45]. Conversely, a lack of trust in screening programs emerged as a critical factor. The 24.4% of participants who expressed distrust (by disagreeing with the statement ‘I trust screening programs’) contrast with some regional data [24, 37], but the finding that it was significantly associated with poor uptake (*p* < 0.001) underscores the need to build patient-provider rapport through culturally sensitive counselling.

## Limitations

This study has several limitations that should be considered when interpreting the findings:

- Cross-Sectional Design: The cross-sectional nature of the study allows for identifying associations, such as between trust in healthcare providers and screening adherence, but it does not permit the determination of causality.
- Sampling Bias: The use of convenience sampling from primary care centers may limit the generalizability of the results to the broader female population of Kuwait. The sample likely overrepresents health-conscious or health-literate women who are already accessing healthcare services.
- Self-Reported Data: The reliance on self-reported data introduces potential bias, as participants may provide socially desirable responses or inaccurately recall sensitive information, such as fear, trust in healthcare providers, or screening history.
- Small Subgroup Sizes: The relatively small sample sizes for certain subgroups, such as those defined by marital status, may affect the reliability and robustness of statistical comparisons, suggesting caution in interpreting these findings.
- Questionnaire Validity and Reliability: Although the questionnaire was revised by experts and piloted, some concerns may still raise about the accuracy of the measurements. Additionally, no statistical assessments of validity or reliability (e.g., internal consistency measures) were reported, limiting a comprehensive evaluation of the questionnaire’s robustness.
- Lack of Comparative Studies: The scarcity of local studies on mammography screening in Kuwait hinders comparative analysis and contextualization of the results within the regional context.

## Recommendations

More population-based studies are needed in Kuwait to ensure better representation and more understanding of the target population. Mixed methods approach (e.g., focus groups) are also needed to explore qualitative drivers of trust, discomfort, and decision-making.

The KNMSP needs to strengthen its outreach via deploying proactive Invitations. This can be achieved by using automated reminders and employer partnerships. Culturally Tailored Education is needed to correct misconceptions about screening age/frequency through community campaigns using relevant local narratives. Finally, utilizing a pre-screening counselling model and training radiographers in patient-centred techniques might improve the significant barriers identified by this study.

## Conclusion

This study highlights critical barriers to mammography screening among Kuwaiti women aged 40–69, including fear of pain, poor awareness despite program familiarity, and systemic gaps in outreach. Only 25.4% of participants underwent screening in the past two years, with 85.6% expressing negative attitudes linked to physical discomfort and sociocultural hesitations.

## Supplementary Information

Supplementary Material 1.

## Data Availability

The datasets used and/or analyzed during the current study are available from the corresponding author on reasonable request.
