# Supplementary material for: Barriers and limitations for undergoing mammography screenings among Kuwaiti women (aged 40–69) attending primary health care centers
Source: BMC Prim Care. 2025 Sep 29;26:295. doi: 10.1186/s12875-025-02971-2 (PMC12482398; doi:10.1186/s12875-025-02971-2)
Supplement: Supplementary file 1 — Supplementary Material 1. [file 12875_2025_2971_MOESM1_ESM.pdf]

Study questionnaire

**Case ID:**	Date of data collection:	Data collector:
Health centre name:	**Governorate:**	

Dear Participant,

This short survey is about the knowledge and attitude towards breast cancer screening. Your participation is voluntary, and you may withdraw from this study if you want to. These answers will be anonymous as they do not require your ID or name. Therefore, we will not identify you in any report coming from this survey. We would appreciate it if you took out your valuable time to answer this survey **(around 15 minutes).**

A. Socio-demographic characteristics.

1.	Age	_ _					
2.	Marital status	☐ Single	☐ Married	☐ Divorced	☐ Widow		
3.	Level of education	☐ Illiterate	☐ Primary or secondary school	☐ High school or diploma	☐ Bachelor's degree	☐ Post-graduate	
4.	Employment status	☐ Government worker	☐ Private sector worker	☐ Student	☐ Home maker	☐ Retired	☐ Unemployed

B. Mammography Screening uptake

1.	Have you ever been invited for breast screening in Kuwait?	☐ Yes			☐ No	
2.	Have you ever had a mammogram?	☐ Yes			☐ No	
3.	If you had a mammogram before, how long has it been since you had your last mammogram?	☐ <12 months	☐ 1-2 years	☐ 3-4 years	☐ > 4 years	☐ Unsure

C. Mammography Screening Awareness

1.	Is there a breast cancer screening program in Kuwait?	☐ Yes		☐ No		
2.	What makes a woman perform mammography? <u>(You can choose more than one option)</u>	☐ It is a routine activity after a certain age	☐ If she has a nipple discharge.	☐ If she feels a lump in her breast	☐ If she feels pain in her breasts	☐ If a relative was diagnosed with breast cancer.
3.	At which age are women **recommended to perform** mammography in Kuwait?	☐ 35	☐ 40	☐ 45	☐ 50	☐ 55
4.	How frequent should mammography screening be performed?	☐ Every 6 months	☐ Every year	☐ Every 2 years	☐ Every 5 years	

D. Barriers to screening

**To which extent do you agree with each of the following statements?**		**Strongly agree**	**Agree**	**Not sure**	**Disagree**	**Strongly disagree**
1.	I feel uncomfortable when my body is being touched during the mammography.	☐	☐	☐	☐	☐
2.	I am afraid of experiencing pain during the mammography screening.	☐	☐	☐	☐	☐
3.	I received conflicting advice regarding the mammography screening program.	☐	☐	☐	☐	☐
4.	I see the course of breast cancer as predetermined	☐	☐	☐	☐	☐
5.	I would rather not know whether something is wrong as a result of the screening.	☐	☐	☐	☐	☐
6.	I am unsure what to expect out of mammography.	☐	☐	☐	☐	☐
7.	The radiation of mammography screening is harmful	☐	☐	☐	☐	☐
8.	I feel obliged through the invitation.	☐	☐	☐	☐	☐
9.	I have trust in the mammography Screening Program.	☐	☐	☐	☐	☐
10.	I have other problems that are more important than a mammography.	☐	☐	☐	☐	☐
